# Nasal Rehabilitation in a Patient After Basal Cell Carcinoma and Radiotherapy: A Case Report

**DOI:** 10.1002/ccr3.70020

**Published:** 2025-01-06

**Authors:** Tamana Barakati, Hedayatullah Ehsan, Omid Humkar, Hassina Shadab, Munir Ahmad Ibrahimkhil

**Affiliations:** ^1^ Faculty of Medicine and Dentistry Queen Mary University of London London UK; ^2^ Medical Sciences Research Center Ghalib University Kabul Afghanistan; ^3^ Faculty of Medicine and Dentistry Kabul University of Medical Sciences "Abu Ali Ibn Sina" Kabul Afghanistan; ^4^ Noman Sadat Institute of Higher Education Kabul Afghanistan; ^5^ Department of Periodontology Kabul University of Medical Science "Abu Ali Ibn Sina" Kabul Afghanistan

**Keywords:** basal cell carcinoma, facial prosthesis, interdisciplinary care, nasal prosthesis, nasal rehabilitation, quality of life

## Abstract

Nasal rehabilitation following basal cell carcinoma (BCC) and radiotherapy presents significant challenges due to the intricate balance between aesthetic and functional restoration. This case report discusses the rehabilitation of a 73‐year‐old male who underwent surgical excision and radiotherapy for BCC located on the left ala of the nose. Post‐treatment, the patient experienced dissatisfaction with his facial appearance, negatively impacting his quality of life. This study highlights the critical role of interdisciplinary collaboration in achieving optimal outcomes through prosthetic reconstruction. The process involved careful surgical removal of residual tissue, followed by detailed impression‐taking, modeling, and fabrication of a custom nasal prosthesis. Key considerations included anatomical accuracy, symmetry, and color matching to ensure a natural appearance. Emphasis was placed on patient‐centered care, addressing both physical and psychological aspects. Despite achieving satisfactory results, limitations such as the absence of long‐term follow‐up data were noted. Future research should focus on long‐term prosthesis performance and patient‐reported outcomes to enhance rehabilitation strategies. This case underscores the importance of an integrated approach combining surgical precision, prosthodontic expertise, and psychological support to improve patient satisfaction and quality of life following nasal rehabilitation post‐BCC and radiotherapy.

**Summary**

- Nasal rehabilitation post‐basal cell carcinoma and radiotherapy requires a multidisciplinary approach to address both functional and psychosocial aspects, ultimately enhancing patient outcomes and quality of life.

## 1 Introduction

Basal cell carcinoma (BCC) is the most common type of skin cancer, accounting for approximately 80% of all non‐melanoma skin cancers. It arises from the basal cells in the epidermis and is primarily caused by prolonged exposure to ultraviolet (UV) radiation from the sun or tanning beds. BCC is typically slow‐growing and rarely metastasizes, but it can cause significant local destruction if not treated promptly. The treatment options for BCC include surgical excision, Mohs micrographic surgery, cryotherapy, photodynamic therapy, and topical medications. Among these, surgical excision remains the gold standard due to its high cure rates and ability to provide a histopathological examination of the margins [1, 2, 3, 4, 5].

Radiotherapy is often employed as an adjunctive treatment for BCC, particularly in cases where surgical margins are positive or inoperable tumors are present. It is also a viable option for patients who are not suitable candidates for surgery due to age, comorbidities, or anatomical location of the tumor. Radiotherapy involves the use of high‐energy X‐rays or other radiation particles to destroy cancer cells. While effective, it can lead to acute and long‐term side effects such as skin changes, fibrosis, and increased risk of secondary malignancies [2, 6, 7].

Nasal rehabilitation following the surgical and radio therapeutic treatment of BCC poses unique challenges due to the importance of the nose in facial aesthetics and function. Reconstruction aims to restore the form and function of the nose, often requiring a multidisciplinary approach involving oncologists, surgeons, and prosthodontists. This process not only addresses the physical defects but also significantly impacts the patient's psychological well‐being and quality of life [8, 9, 10].

The nose, being a prominent feature of the face, plays a crucial role in facial aesthetics and function. Hence, nasal rehabilitation following surgical and radio therapeutic treatment of BCC presents unique challenges. Reconstruction seeks to regain both the shape and functionality of the nose, typically necessitating a collaborative effort among oncologists, surgeons, and prosthodontists. Addressing the physical defects is essential, but equally important is managing the psychological impact on the patient's well‐being and quality of life [1, 2, 3, 4, 5, 6, 8, 9, 10, 11, 12, 13, 14, 15, 16].

Nasal prostheses are an effective option for patients requiring reconstruction after BCC treatment. The process involves creating a customized prosthesis that matches the patient's skin color and anatomical features. Detailed impression‐taking, modeling, and fabrication are necessary to achieve a natural appearance and functional outcome. The goal is to provide an aesthetically pleasing result that improves the patient's self‐esteem and quality of life [2, 17, 18, 19].

In this study, we present a case of a patient who underwent surgical excision and radiotherapy for BCC of the left ala of the nose. Post‐treatment, the patient expressed significant dissatisfaction with his facial appearance, impacting his self‐esteem and overall quality of life. This scenario highlights the importance of addressing both physical and psychological aspects in post‐oncological rehabilitation.

The rehabilitation process involved the removal of the residual portion of the left ala nasal to create a defect suitable for prosthetic reconstruction. Following this, a customized nasal prosthesis was fabricated, considering anatomical accuracy, symmetry, and color matching. Continuous collaboration between the patient, the surgeon, and the prosthodontist was crucial to ensure that the prosthetic solution met the patient's aesthetic and functional needs [8].

Despite the successful outcomes, the case also illustrated certain limitations, such as the absence of long‐term follow‐up data and subjective patient‐reported outcomes. Future research should focus on evaluating the long‐term performance of nasal prostheses and their overall impact on patient‐reported outcomes to improve rehabilitation strategies further.

Through this case report, we aim to elucidate the comprehensive approach to nasal rehabilitation post‐basal cell carcinoma and radiotherapy, emphasizing the importance of addressing both functional and psychosocial aspects to improve patient outcomes and quality of life.

### 1.1 Case History/Examination

A 73‐year‐old male patient with an education degree diploma and married status presented with a moderate socioeconomic background. Psychologically, he appeared indifferent. His chief complaint centered on dissatisfaction with his facial appearance, expressing feelings of ugliness and annoyance. This concern highlights a predominantly static issue affecting his self‐perception and quality of life. In his medical history, the patient had basal cell carcinoma located in the left ala of the nose. A nodular ulcer lesion on the skin at the left ala nasal margin necessitated surgical intervention. The excised nodular lesion from the left ala of the nose was sent for histopathological examination, which confirmed the diagnosis of basal cell carcinoma with clear margins, indicating successful removal. Following the removal of the lesion, the patient underwent 17 sessions of radiotherapy as part of the treatment plan.

**FIGURE 1:**
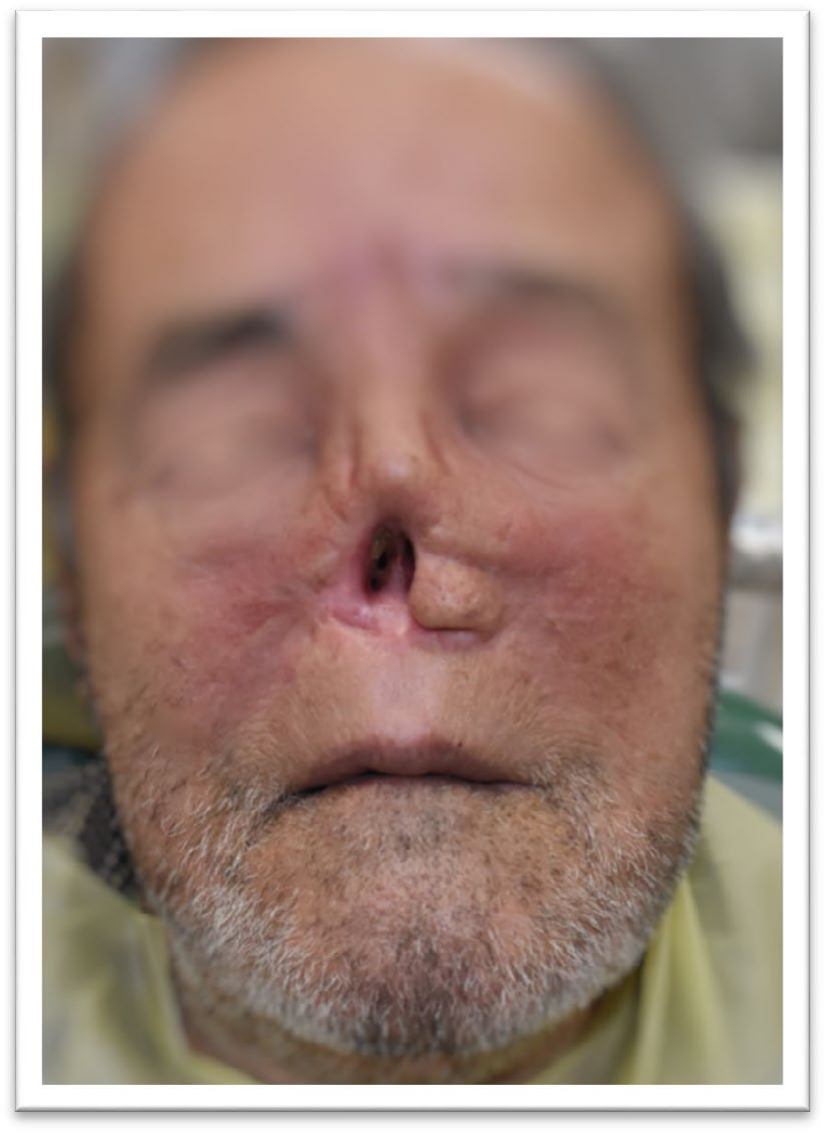
Pre‐Surgical Condition: The patient before surgical excision, showing the nodular ulcer lesion on the left ala of the nose.

After two years' post‐surgery and radiotherapy, during the patient's revisit for nose rehabilitation, a facial examination revealed an ovate facial shape, equal facial proportion, and competent lip function. Functional assessment indicated normal jaw movement with a maximum opening of 40 mm without clicking, crepitus, or deviation during mouth opening and closing. No limitations in movement, pain, or tenderness were noted in the face, and lymph nodes were not palpable. Muscle palpation revealed no tenderness. However, a residual portion of the left ala nasal remained, rendering the nose defect unfavorable for prosthetic reconstruction (Figure 1). Hence, it was recommended and subsequently accepted by the surgeon to remove this unsupportive portion to facilitate fitting a nasal prosthesis. Despite the patient's advanced age and previous history of basal cell carcinoma treated with radiotherapy, the desire for a nasal prosthesis remained, prompting this additional surgical intervention

**FIGURE 2:**
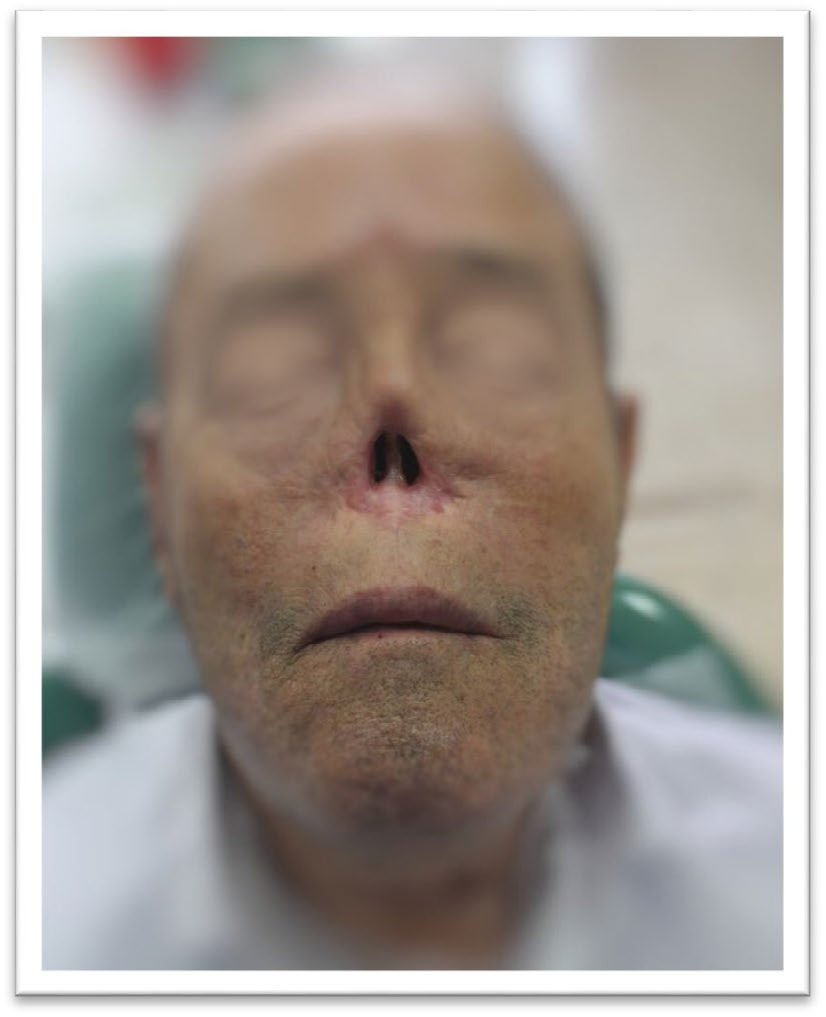
Post‐Surgical Defect: The patient's nasal defect after the surgical removal of basal cell carcinoma, before radiotherapy.

**FIGURE 3:**
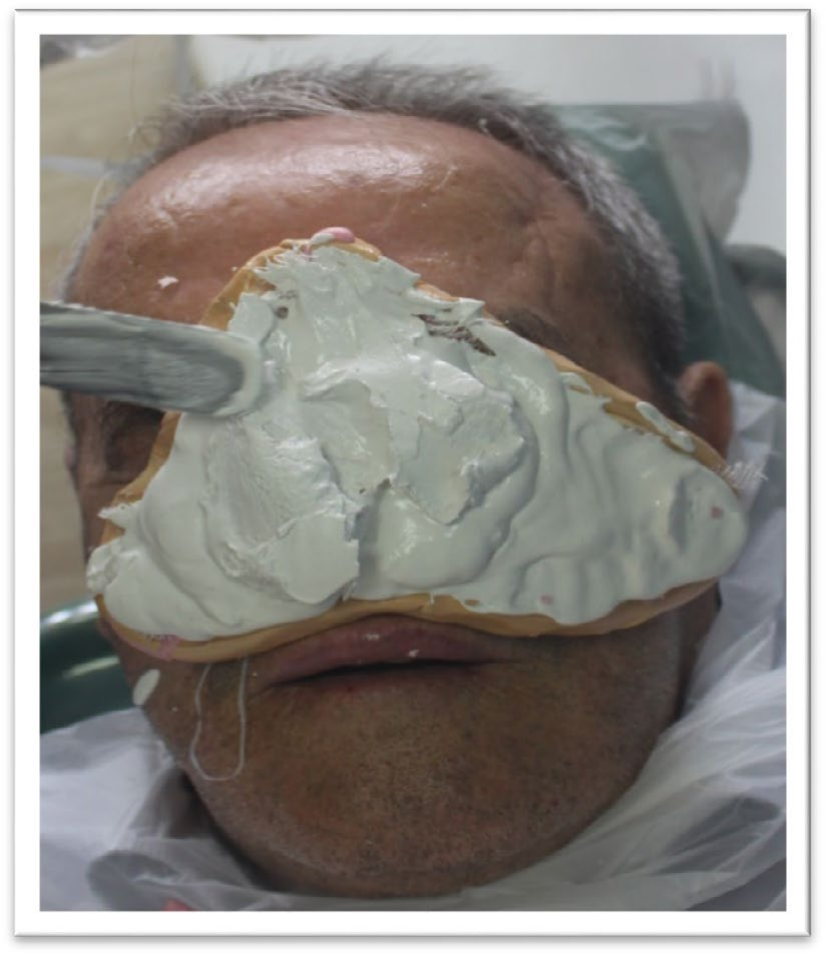
Impression‐taking Process: Alginate material being applied to the patient's face to capture the nasal impression.

Following the successful surgery to remove the left ala nasal, the nose defect became favorable for prosthetic reconstruction (Figure 2). Three months' post‐surgery, with the nose healed and the edema resolved, the patient proceeded to the next step in the rehabilitation process. The initial stage involved taking impressions of the patient's nose using alginate material (Figure 3). This entailed creating a border around the nose, applying Vaseline to the patient's face, and inserting cotton rolls into the nasal cavity for control. Alginate mixed with water, gypsum, and salt was then applied to capture the facial impression. Gas was introduced to aid in the setting, followed by Gibson's application to reinforce the impression. The patient was positioned at a 45° angle for the implantation process and stent placement. Once the alginate and gypsum had set, the patient was asked to perform facial movements to aid in the gentle removal of the prosthesis. After removal, the accuracy of the alginate copy was checked before embedding it in gypsum.

**FIGURE 4:**
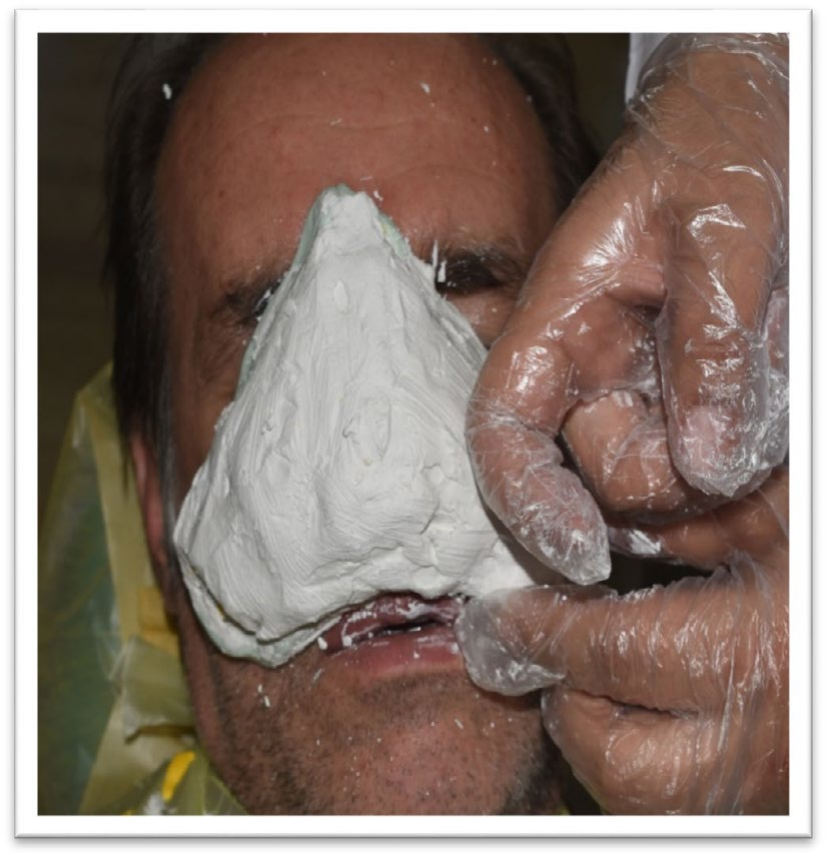
Wax Contouring: The wax model being shaped on the patient's cast to ensure anatomical accuracy and symmetry.

**FIGURE 5:**
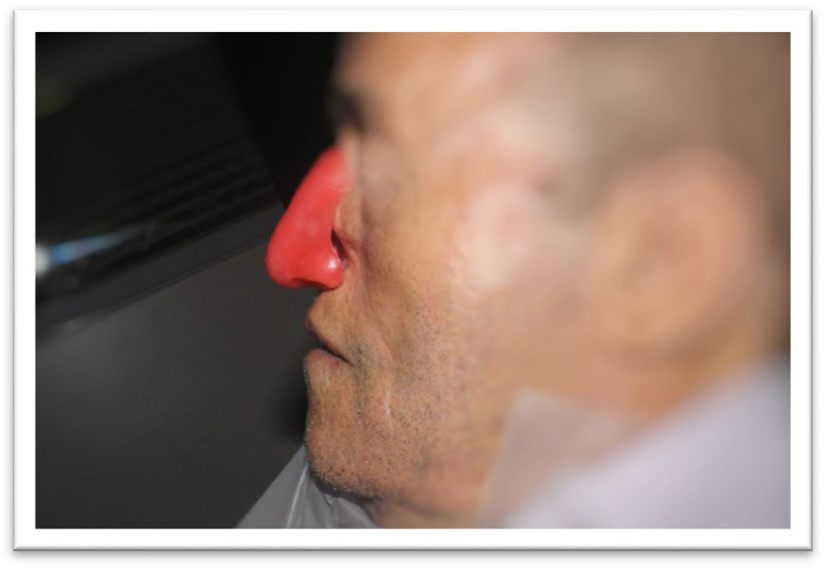
Silicone Injection: Injection of silicone elastomer into the mold with intrinsic coloration to match the patient's skin tone. The prosthesis was prepared following radiotherapy.

**FIGURE 6:**
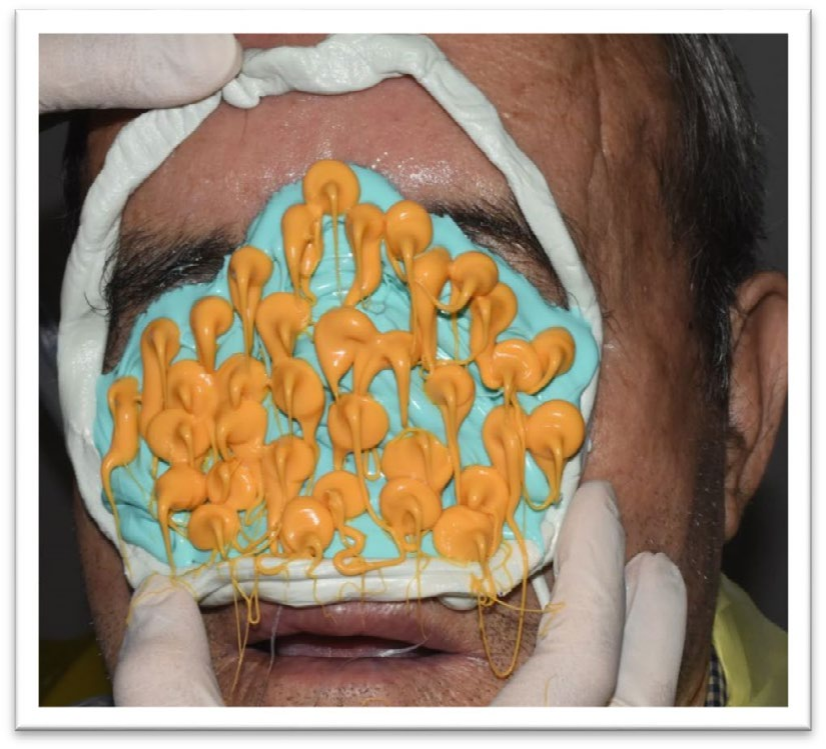
Final Nasal Prosthesis: The completed nasal prosthesis, post‐coloration and trimming, ready for fitting after the healing period following radiotherapy.

Subsequently, it was decided to also take an impression from the patient's son as the patient desired the replacement nose to resemble his son's. The same procedure was repeated for the son's facial impression, resulting in two casts—one from the patient and one from the patient's son—for further modeling and shaping of the prosthetic nose (Figure 4). Wax was then used to contour the nose on the cast, ensuring symmetry and anatomical correctness. Measurements were taken from the stent to determine the length, height, and other dimensions concerning the patient's nose and ear length for proportional accuracy. However, upon pattern application, discrepancies between the pattern and the patient's face were noted, prompting the decision to take a new impression using polymer material (Figure 5). This involved creating a border around the patient's face, applying Vaseline, injecting polymer, and setting it with gypsum (Figure 6). Patient cooperation, relaxation, and breathing through the mouth were crucial during impression‐taking. After removal, corrections were made and the pattern was refined for subsequent application on the patient's face during the next visit.

**FIGURE 7:**
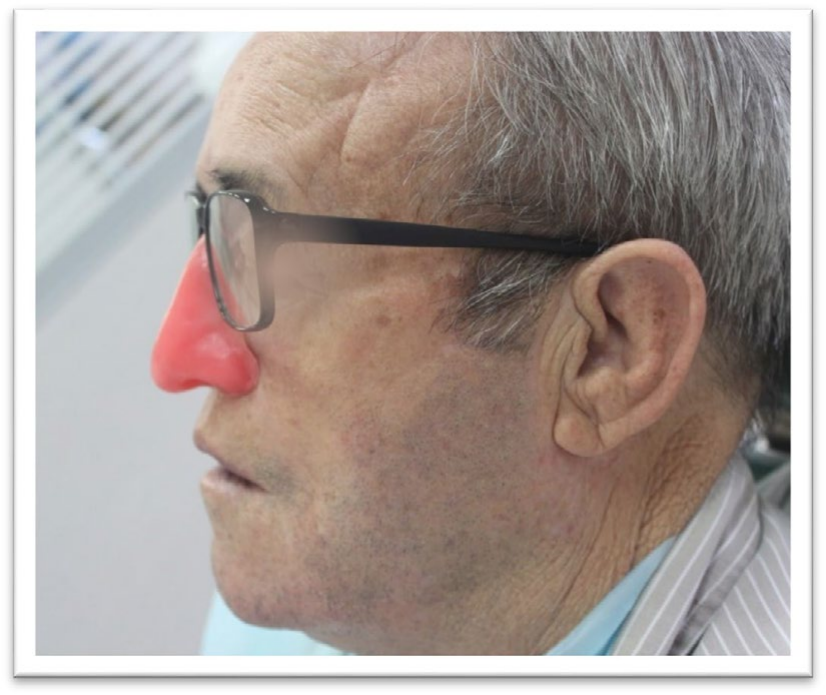
Fitted Prosthesis: The nasal prosthesis fitted to the patient's face, demonstrating the aesthetic restoration achieved.

After ensuring the wax pattern's adequacy from various angles and securing retention with glass and nasal undercuts, the patient's agreement on size and shape prompted progression to the next steps (Figure 7). The wax pattern was meticulously flossed and eliminated to prepare for silicone injection. Coloration of the nasal prosthesis was a crucial aspect of the process. Cleaning the flask and applying Vaseline preceded using silicone elastomer for its skin‐like texture, ease of coloring, and patient suitability. Intrinsic coloration involved mixing silicone with different shades to match the patient's skin tone, incorporating colors such as blue, black, red, brown, white, yellow, and pink. This mixture was then injected into the flask, with additional coloration applied to the margins for consistency. The flask was sealed and left to set for 24 h to complete the process.

**FIGURE 8:**
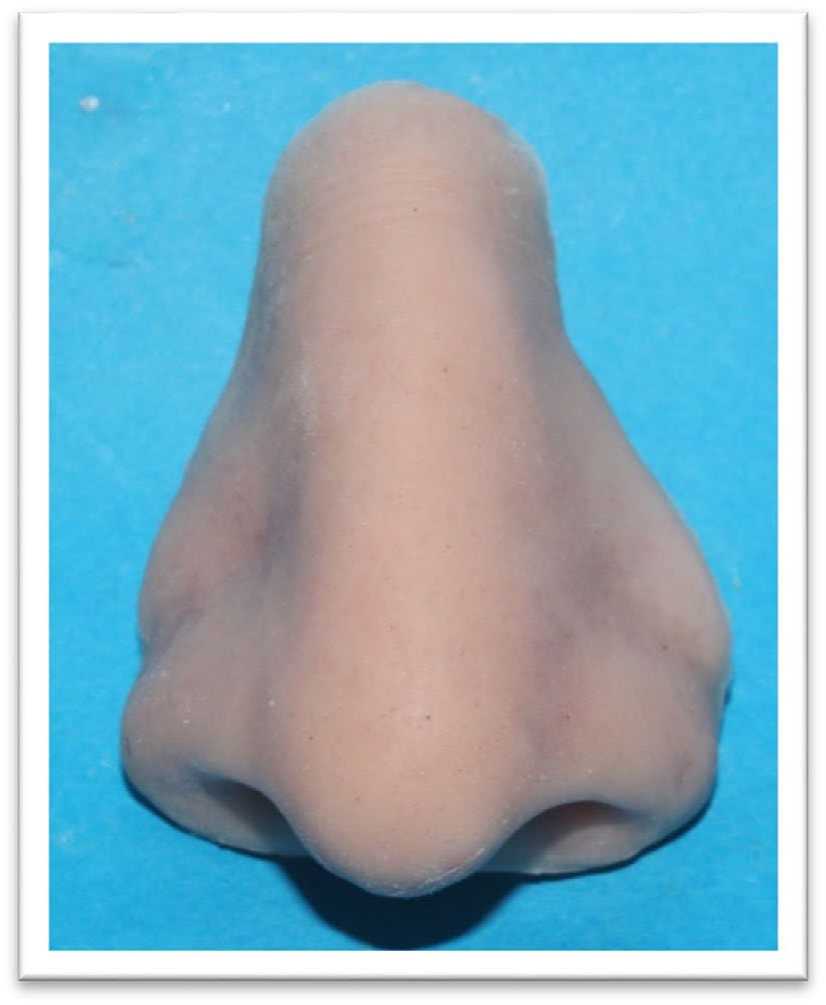
Removal of Prosthesis from Flask: The nasal prosthesis being removed from the flask and the margins being trimmed with scissors after 24 h of setting.

**FIGURE 9:**
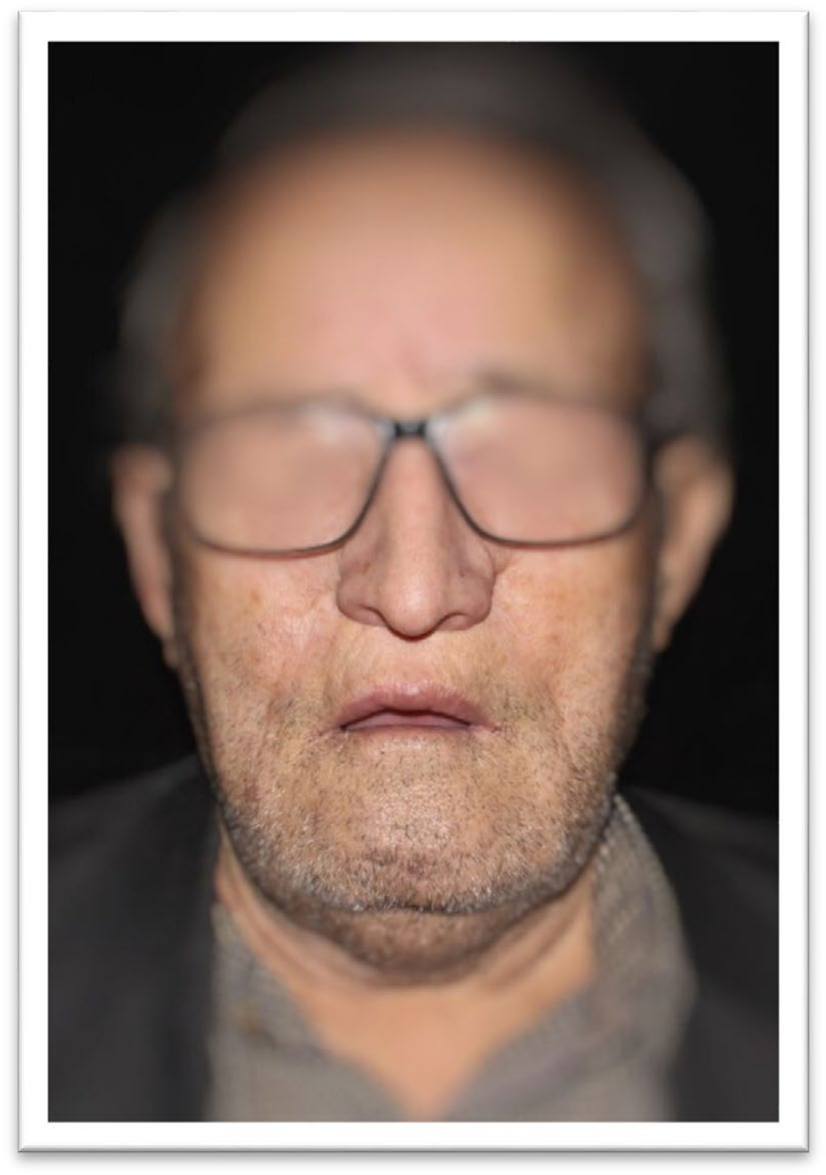
Retention Mechanism: Close‐up of the prosthesis secured with undercuts, skin adhesive, and eyeglasses for retention.

After the 24‐h setting period, the nose prosthesis was removed from the flask and the margins were trimmed with scissors (Figure 8). Once again, it was fitted to the patient's face for adaptation assessment, which proved satisfactory. Additional extrinsic coloration was necessary to match the patient's skin tone, addressing areas of concern such as CAG, MAG, and spots. After applying the color, the prosthesis was left to dry for an hour before being securely placed on the patient's face (Figure 9). Retention was achieved through engaged undercuts, skin adhesive, and eyeglasses, ensuring a secure fit and comfort for the patient.

Based on the recommendations outlined in the article, the patient was advised to remove the nose prosthesis during nighttime rest and activities involving strenuous exertion such as exercise or running. Washing the prosthesis should be done sparingly, preferably once a week, and soap should be avoided. Additionally, exposure to ultraviolet light should be minimized to preserve the integrity of the prosthesis. During activities, removing the prosthesis from the face is recommended to ensure its longevity and proper maintenance.

## 2 Methods

The process began with taking impressions of the patient's nasal defect using alginate material, ensuring an accurate mold for prosthetic reconstruction. The patient expressed a desire for the replacement nose to resemble his son's, so an impression from the patient's son was also taken.

The wax pattern of the nose was meticulously contoured on the cast, ensuring symmetry and anatomical correctness. Measurements were taken for proportional accuracy. Silicone elastomer was used to form the prosthesis due to its skin‐like texture and ease of coloring. Coloration involves intrinsic and extrinsic color matching to ensure a natural appearance. After the 24‐h setting period, the prosthesis was fitted to the patient's face, and additional coloration was applied to match the patient's skin tone.

The prosthesis was fixed using a combination of medical adhesives and natural undercuts, allowing for daily wear and removal. Although Osseo‐integrated implants were considered for better retention, they were not suitable due to the patient's previous radiotherapy.

## 3 Results

The case presented exemplifies the successful rehabilitation journey of a 73‐year‐old male patient post‐basal cell carcinoma excision and radiotherapy, culminating in the restoration of facial aesthetics and functionality through a customized nasal prosthesis. This comprehensive approach involving interdisciplinary collaboration and patient‐centric care highlights key insights and considerations for nasal reconstruction in similar clinical contexts.

Through meticulous surgical planning and prosthetic fabrication, the patient's dissatisfaction with facial appearance was effectively addressed, leading to improvements in self‐perception and quality of life. The removal of the residual portion of the left ala nasal facilitated optimal prosthesis adaptation, emphasizing the importance of surgical precision in achieving desirable outcomes. Psychosocial factors played a significant role throughout the rehabilitation process, underscoring the importance of holistic patient care beyond physical restoration. Addressing patient concerns and providing ongoing support is crucial for enhancing overall well‐being and treatment success.

Attention to detail in impression‐taking, modeling, and color matching ensured the creation of a natural‐looking and comfortable nasal prosthesis. Iterative refinement based on patient feedback exemplified the patient‐centered approach adopted, contributing to high levels of satisfaction and acceptance. Despite the successful rehabilitation achieved in this case, it is essential to acknowledge the limitations, including the absence of long‐term follow‐up data and subjective patient‐reported outcomes. Future research endeavors should prioritize evaluating long‐term prosthesis performance and patient satisfaction to inform evidence‐based practice and optimize outcomes in similar clinical scenarios.

The successful rehabilitation of the patient following basal cell carcinoma excision and radiotherapy culminated in the fabrication and fitting of a customized nasal prosthesis. This comprehensive approach, involving interdisciplinary collaboration and patient‐centric care, led to improvements in self‐perception and quality of life. The patient was advised to remove the nose prosthesis during nighttime rest and strenuous activities, wash it sparingly, and minimize exposure to ultraviolet light. In conclusion, this case report underscores the significance of interdisciplinary collaboration, patient‐centered care, and meticulous attention to detail in achieving successful nasal rehabilitation post‐basal cell carcinoma and radiotherapy. By addressing both functional and psychosocial aspects, we aim to enhance patient outcomes and improve the overall quality of life in individuals undergoing similar journeys of nasal reconstruction.

## 4 Discussion

The case presented illustrates the successful rehabilitation of a patient following basal cell carcinoma excision and subsequent radiotherapy, culminating in the fabrication and fitting of a customized nasal prosthesis. This discussion delves into the clinical implications, challenges encountered, and considerations for future management.

The interdisciplinary approach involving surgical, prosthodontic, and patient‐centric care was pivotal in addressing both functional and aesthetic concerns. Collaboration among specialists facilitated comprehensive evaluation, treatment planning, and execution, ensuring optimal outcomes tailored to the patient's needs and desires [10, 17]. Psychological factors significantly influenced the patient's journey, as evidenced by his dissatisfaction with facial appearance and negative self‐perception [17]. Addressing psychosocial aspects alongside physical rehabilitation is essential for holistic patient care and improving quality of life post‐oncological treatment [19, 20, 21].

In addition to traditional nasal prosthesis fabrication, there are several other methods of nasal reconstruction available for patients who have undergone excision of basal cell carcinoma. Surgical reconstruction is a common approach, where local or distant flaps of skin and cartilage are used to rebuild the nose [3, 19, 22, 23]. Surgical methods can provide a permanent solution and avoid the need for external prostheses. However, surgical reconstructions are complex, particularly in cases where patients have undergone radiotherapy, which can compromise tissue healing. Additionally, achieving both functional and aesthetic success through surgery often requires multiple procedures and long recovery periods [18, 24, 25, 26, 27].

Emerging technologies such as 3D printing offer promising alternatives for nasal reconstruction. In recent years, 3D‐printed prostheses have gained traction due to their precision and ability to customize designs that perfectly match the patient's anatomical structure [23, 28]. This technology allows for exact replicas of the patient's nose to be created using imaging and 3D modeling. While 3D printing provides excellent aesthetic outcomes, particularly with advancements in materials that mimic the texture and color of human skin, it remains less accessible due to cost and limited availability in clinical settings. Moreover, the durability and long‐term functionality of 3D‐printed prostheses are still being evaluated [23, 28].

In this case, a custom‐made prosthesis was chosen over surgical and 3D‐printed alternatives due to the patient's previous history of radiotherapy and advanced age. Surgical options were considered less ideal due to potential healing complications, and 3D printing was not readily available. The prosthetic approach involved detailed impression‐taking, wax modeling, and color matching, with iterative adjustments based on patient feedback to ensure a satisfactory outcome [17, 23, 28, 29]. This method remains highly effective in cases where surgical reconstruction is not feasible, providing patients with a non‐invasive solution that can be easily maintained and replaced as needed.

Long‐Term Follow‐up: One limitation of this case is the absence of long‐term follow‐up data regarding the durability of the prosthesis and the patient's satisfaction. To address this gap, future assessments could be conducted via telephone return visits and questionnaire surveys. These tools would enable clinicians to gather valuable feedback on the long‐term functionality of the prosthesis, its impact on the patient's psychosocial well‐being, and any maintenance challenges faced. Implementing follow‐up surveys at intervals such as 6 months and 12 months' post‐rehabilitation could provide deeper insights into patient‐reported outcomes, including prosthesis comfort, daily usability, and psychological effects. By utilizing remote follow‐up methods, it becomes feasible to monitor patient progress without imposing the logistical burden of in‐person clinic visits [21, 30].

Coloration of the nasal prosthesis was a critical aspect of the rehabilitation process, requiring intrinsic and extrinsic color matching to achieve a natural appearance [29, 30]. Patient education regarding prosthesis maintenance, including cleaning, storage, and UV protection, is essential for prolonging prosthesis longevity and preserving aesthetic integrity [22, 30].

In summary, nasal rehabilitation after basal cell carcinoma and radiotherapy requires a comprehensive approach that considers both functional and psychosocial factors to improve patient outcomes. While surgical reconstruction and 3D printing present viable alternatives to prosthetic solutions, each method has its limitations and must be carefully evaluated based on the patient's medical history and personal needs. This case report highlights the significance of teamwork, patient‐focused care, and careful attention to detail in achieving effective nasal reconstruction and enhancing quality of life.

## Author Contributions

All authors have made significant contributions to the conception, design, execution, and interpretation of the study. Prof. Dr. Tamana Barakati led the surgical and prosthodontic procedures, and Asst. Prof. Dr. Hedayatullah Ehsan led to the data collection, and manuscript preparation. Rest of the co‐authors contributed to patient care, data analysis, and manuscript review. All authors have approved the final manuscript.

## Ethics Statement

This case was conducted at the university hospital of Tehran University of Medical Sciences under the supervision of professors from the prosthetics department. All procedures followed were in accordance with the ethical standards of the responsible committee on human experimentation and with the Helsinki Declaration. Informed written consent was obtained from the patient for participation in the study, including the use of all personal information and images for research purposes.

## Data Availability

Access to the data is available upon request, subject to approval by the research committee and correspondence author this study (Dr. Hedayatullah Ehsan). Interested parties may obtain access by submitting a polite request to the designated contact person. We are committed to facilitating access to the data while ensuring compliance with ethical and privacy considerations.
